# The relationship between professional self-concept and career decision-making difficulties among postgraduate nursing students in China: the mediating role of career decision-making self-efficacy

**DOI:** 10.3389/fpsyg.2023.1198974

**Published:** 2023-07-31

**Authors:** Yaping Bi, Shaoyu Mou, Ge Wang, Mingyan Liao

**Affiliations:** ^1^Department of Hematology, The First Affiliated Hospital of Chongqing Medical University, Chongqing, China; ^2^School of Nursing, Chongqing Medical University, Chongqing, China

**Keywords:** postgraduate nursing students, career decision-making difficulties, career decision-making self-efficacy, professional self-concept, mediating role, social cognitive career theory

## Abstract

**Background:**

In the context of a global shortage, uneven distribution, and structural imbalance of nursing talent, postgraduate nursing students must make appropriate decisions about their careers not only for the nursing profession but also for society as a whole. However, little research has been reported on the current status and factors influencing career decision-making difficulties among postgraduate nursing students.

**Objectives:**

Exploring the mediating role of career decision-making self-efficacy between professional self-concept and career decision-making difficulties among postgraduate nursing students in China based on the social cognitive career theory.

**Methods:**

276 postgraduate nursing students from 25 universities in seven administrative regions of China were selected by stratified random sampling. Data were collected with the Career decision-making difficulties Questionnaire, Career Decision-making Self-Efficacy Scale, and Nursing Professional Self-concept Scale through an online survey, and were analyzed by univariate analysis, correlation analysis, multiple linear regression analysis, and PROCESS macro.

**Results:**

The score for career decision-making difficulties was 2.84 (SD = 0.54). Professional self-concept (*r* = −0.496, *p* < 0.01) and career decision-making self-efficacy (*r* = −0.551, *p* < 0.01) were negatively associated with career decision-making difficulties. Career decision-making self-efficacy played a partial mediating role between professional self-concept and career decision-making difficulties (*p* < 0.01), with the mediating effect (Effect Value = −0.253, Bootstrap 95% CI: −0.349, −0.156) accounting for 53.82% of the total effect.

**Conclusion:**

The high scores of career decision-making difficulties among postgraduate nursing students demand widespread attention. Nursing educators need to develop a complete and standardized career counseling curriculum for postgraduate nursing students, and should pay attention to the cultivation and development of positive professional self-concept and career decision-making self-efficacy of postgraduate nursing students to reduce their career decision-making difficulties and help them make effective career decision-making.

## 1. Introduction

A career has a significant impact on an individual’s life. It is not only a means of making a living but also a way of self-development and self-realization. Career decision-making difficulties refer to the difficulties that occur when an individual does not know what occupation to pursue or chooses one of several occupations when facing the final decision in the process of a career decision, including three groups of difficulties: lack of readiness, lack of information and inconsistent information, among which lack of readiness occurs before the career decision process begins, the other two tend to occur after the career decision-making process has begun (Gati et al., 1996; Lipshits-Braziler et al., 2016).

Career decision-making difficulties often have a negative impact on nursing students, making them unable to make a career decision at the right time or causing them make the wrong career decision, which leads to lower professional identity, higher job burnout and turnover intention after work (Gan et al., 2020; Wang, 2020; Ren et al., 2021). Considering that postgraduate nursing students, as high-level nursing talents, are the mainstay of the nursing industry, at the same time, in the context of a global shortage, uneven distribution, and structural imbalance of nursing talent (Hou et al., 2014; WHO, 2020), postgraduate nursing students must make appropriate decisions about their careers not only for the nursing profession but also for society as a whole (Ni et al., 2022). In addition, the value of providing structured courses to help medical students make career decisions has been recognized (Navarro et al., 2011; Park, 2015; Howse et al., 2017). However, the core courses of postgraduates nursing education in China mainly include Advanced Health Assessment, Advanced pathophysiology, Advanced pharmacotherapy, Evidence-based nursing, Nursing Theory, Nursing Research, Nursing pedagogy, etc., and there is a lack of a complete and standardized career counseling curriculum system (Sun et al., 2015; Zhang et al., 2019; Wang et al., 2020). A better understanding of postgraduate nursing students’ career decision-making difficulties and its related influencing factors will provide a reference for designing targeted interventions to alleviate postgraduate nursing students’ career decision-making difficulties. However, few studies have focused on the career decision-making difficulties of postgraduate nursing students (Zhou et al., 2021).

Professional self-concept refers to the individual’s self-evaluation of his or her professional knowledge, value, and skills (Kelly, 1992), which plays an important core and driving role in the individual’s career choice and career development (Yang et al., 2008). Studies have shown that nurses with higher professional self-concept have a stronger professional identity, higher job satisfaction, and lower job burnout and turnover intention (Goliroshan et al., 2021; Li et al., 2021). For nursing students, those with more positive professional self-concept have more adequate preparation for career selection, more positive career selection behavior, and more smooth career selection process (Lee, 2019). However, the mechanism of the influence of professional self-concept on the career decision-making and development of postgraduate nursing students is still unknown.

Self-efficacy is an individual’s belief in his or her ability to organize and execute a series of actions to achieve a specific goal. According to the social cognitive career theory (SCCT) (Lent et al., 1994), career decision-making self-efficacy is the application of self-efficacy in the career field, that is, an individual’s belief in his or her ability to engage in career decision-making activities (such as collecting career information or selecting career goals) (Betz et al., 1996). Taylor and Betz (1983) proposed that the career decision-making self-efficacy can explain the differences in individual career decision-making difficulties. Relevant studies also showed that career decision-making self-efficacy is negatively correlated with career decision-making difficulties (Penn and Lent, 2018; Santos et al., 2018), that is, the stronger the individual’s confidence in choosing a career, the lower the degree of career decision-making difficulties, and the more effective career decision-making can be made. At the same time, studies have reported that career decision-making self-efficacy is positively correlated with professional self-concept (Lv et al., 2014).

In conclusion, as previous studies have shown, both professional self-concept and career decision-making self-efficacy can affect individuals’ career decision-making. Moreover, according to SCCT (Lent et al., 1994), individual differences, environmental factors, and individual behavior will affect the confidence of the ability of individuals to implement occupation-related tasks and activities through interaction, thereby affecting the process of individual career choice and shaping occupational behavior. However, so far, no studies have examined the comprehensive effect of professional self-concept and career decision-making self-efficacy on the generation of career decision-making difficulties in postgraduate nursing students, and whether career decision-making self-efficacy plays a mediating role between professional self-concept and career decision-making difficulties. SCCT has been applied to several disciplines (Gao et al., 2021), but it has not been used as a conceptual framework to understand the career decisions and development of postgraduate nursing students.

Therefore, the objectives of this study were as follows: (1) to determined what major difficulties impair the career decision-making process among postgraduate nursing students, as measured by career decision-making difficulties, (2) to explore the influence of professional self-concept on the career decision-making difficulties among postgraduate nursing students, and (3) to clarify the mediating role of career decision-making self-efficacy between professional self-concept and career decision-making difficulties among postgraduate nursing students based on SCCT.

## 2. Methods

### 2.1. Study design and participants

This study was designed as a cross-sectional study. From April 2022 to May 2022, considering the representativeness of the sample, a stratified random sampling method was conducted to select 276 postgraduate nursing students from 25 universities in East China (seven universities), South China (four universities), North China (four universities), Central China (two universities), Northeast China (two universities), Northwest China (two universities), and Southwest China (four universities) according to the geographical distribution of China as the study participants. Eligible participants had to be full-time enrolled postgraduate nursing students who volunteered for this survey. The exclusion criteria for this study included: ① postgraduate nursing students who are part-time education graduate students or are employed and ② off work due to medical leave or personal leave during the investigation period. The mean age of the 276 participants was 24.75 (SD = 2.85) years, with a range from 21 to 35 years old. The majority were female (92.03%). Among them, 89 (32.24%) were in their first grade, 120 (43.47%) in their second grade, and 67 (24.29%) in their third grade (as shown in Table 1).

**Table 1:** Participants characteristics and differences of career decision-making difficulties among groups (*n*=276).

Variable	Group	Frequency (percentage)	Career decision-making difficulties (mean ± SD)	*t / F*	*p*
Gender	Male	22 (7.97)	2.59 ± 0.55	−2.270	0.024
	Female	254 (92.03)	2.86 ± 0.53		
Age (years)	<25	161 (58.33)	2.89 ± 0.53	3.240	0.041
	25~29	88 (31.88)	2.81 ± 0.55		
	≥30	27 (9.79)	2.62 ± 0.50		
Registered residence	Rural	158 (57.24)	2.85 ± 0.51	0.433	0.665
	Urban	118 (42.76)	2.82 ± 0.58		
Grade	1	120 (43.47)	2.82 ± 0.53	0.277	0.759
	2	89 (32.24)	2.82 ± 0.59		
	3	67 (24.29)	2.88 ± 0.48		
Degree type	academic degree	81 (29.34)	2.80 ± 0.52	−0.720	0.472
	professional degree	191 (70.66)	2.85 ± 0.55		
Employment experience	Yes	86 (31.16)	2.72 ± 0.52	2.429	0.016
	No	190 (68.84)	2.89 ± 0.54		
Experience in a career development program	Yes	51 (18.47)	2.60 ± 0.58	−3.552	0.000
	No	225 (81.53)	2.89 ± 0.52		

The sample size was calculated according to the principle of Kendall estimation of sample size (Ni et al., 2010). This demonstrated that the sample size was 5 ~ 10 times that of the independent variables. This study contained 20 independent variables. Considering a 20% sample loss rate, the minimum sample size was 120 cases. 276 valid questionnaires were finally collected, with a valid recovery rate of 95.17%, which satisfied the minimum sample size required for this study.

### 2.2. Measurements

#### 2.2.1. Participants’ sociodemographic characteristics

The sociodemographic characteristics of the participants included gender, age, registered residence, grade, degree type, employment experience before enrolling in postgraduate, and experience in a career development program.

#### 2.2.2. Career decision-making difficulties

Career decision-making difficulties were measured using a Chinese version of the Career Decision-making Difficulty Questionnaire developed by (Wu et al., 2016). The questionnaire consists of 36 items across three domains: Lack of Readiness (nine items), Lack of Information (fourteen items), and Inconsistent Information (three items). The responses were provided on a 5-point Likert scale ranging from (1) “strongly disagree” to (5) “strongly agree.” A higher score indicates higher career decision-making difficulties. The questionnaire’s Cronbach’s alpha was 0.816. In this study, Cronbach’s alpha was 0.933.

#### 2.2.3. Career decision-making self-efficacy

Career decision-making self-efficacy was measured using a Chinese version of the Career Decision-making Self-efficacy scale revised by Long (2003). The scale consists of 25 items across five domains: self-appraisal (five items), goal selection (five items), gathering information (five items), planning (five items), and problem-solving (five items). The responses were provided on a 5-point Likert scale ranging from (1) “not confident at all” to (5) “completely confident.” A higher score indicates higher career decision-making self-efficacy. The scale’s Cronbach’s alpha was 0.89. In this study, Cronbach’s alpha was 0.969.

#### 2.2.4. Nursing professional self-concept

Nursing professional self-concept was measured using a scale developed by professor Arthur (Yang et al., 2008). The scale consists of 30 items across five domains: flexibility (7 items), skills (5 items), leadership (4 items), satisfaction (9 items), and communication (5 items). The responses were provided on a 4-point Likert scale ranging from (1) “strongly disagree” to (5) “strongly agree.” A higher score indicates a more positive nursing professional self-concept. The scale’s Cronbach’s alpha was 0.84. In this study, Cronbach’s alpha was 0.896.

### 2.3. Data collection procedures

The person in charge of the survey was appointed in the selected universities to inform them of the purpose of this study and the inclusion and exclusion criteria of the study subjects, ensure their full understanding and obtain their cooperation, and then they were responsible for mobilizing and instructing the postgraduate nursing students in their universities to fill in the questionnaire. The electronic questionnaires were distributed online through the “Wen Juan Xing” web platform, and the research participants filled out the questionnaires anonymously by opening the web link or scanning the WeChat QR code. The questions were all compulsory, and the participants could only submit the questionnaires after completing all the questions independently. The validity and completeness of the completed questionnaires were verified one by one, and the questionnaires that did not meet the completion requirements were excluded.

### 2.4. Data analysis

The Statistical Package for the Social Sciences (SPSS) software (v26) (IBM Corp., Armonk, NY, United States) was used in all analyses. We calculated descriptive statistics for sociodemographic information, professional self-concept, career decision-making self-efficacy, and career decision-making difficulties. We used a *t*-test or an analysis of variance to evaluate differences among the participants in terms of career decision-making difficulties, and Pearson’s r was calculated to test correlations among career decision-making difficulties, professional self-concept, and career decision-making self-efficacy among nursing graduate students. Multiple linear regression analysis was used to explore the mediating effects of professional self-concept, career decision-making self-efficacy, and career decision-making difficulties. Finally, a nonparametric resampling method (5,000 iterations) was applied by running the PROCESS plugin in the SPSS Macro to test the statistical significance of the mediating effect. In all analyses, statistically significant was set at *p*-value < 0.05.

### 2.5. Ethical considerations

This study protocol was approved by the ethics committee of The First Affiliated Hospital of Chongqing Medical University (No. 2022-10) and conformed to the Helsinki Declaration. Via the web, We provided brief purposes and procedures of the study to potential participants, who were also assured that participation was completely voluntary.

## 3. Results

### 3.1. Career decision-making difficulties and its differences among groups

The mean scores of career decision-making difficulties are listed in Table 2. The total mean score of career decision-making difficulties was 2.84 (SD = 0.54). For the three dimensions of career decision-making difficulties, lack of readiness had the highest score of 2.90 (SD = 0.58), and, lack of information showed the lowest score of 2.76 (SD = 0.59).

**Table 2:** Descriptive statistics and correlation analysis between variables (*n* = 276).

	Mean ± SD	1	2	3	4	5	6
1. CDDQ	2.84 ± 0.54	1					
2. LR	2.90 ± 0.58	0.916*	1				
3. LI	2.76 ± 0.59	0.965*	0.828*	1			
4. II	2.88 ± 0.54	0.956*	0.822*	0.883*	1		
5. PSC	2.72 ± 0.36	−0.496*	−0.415*	−0.500*	−0.477*	1	
6. CDMSE	3.32 ± 0.64	−0.551*	−0.441*	−0.564*	−0.536*	0.639*	1

CDDQ, career decision-making difficulties; LR, lack of readiness; LI, lack of information; II, inconsistent information; PSC, professional self-concept; CDMSE, career decision-making self-efficacy. *p < 0.01.

As presented in Table 1, female, younger students, students who were never employed before enrolling in postgraduate education and did not have experience in a career development program had significantly higher scores for career decision-making difficulties (all *p < 0.05*). Registered residence, grade, and degree type were not significantly associated with career decision-making difficulties.

### 3.2. Correlation analysis

As listed in Table 2, correlation analysis showed that career decision-making self-efficacy had a significant negative correlation with career decision-making difficulties (*r* = −0.551, *p* < 0.01), and professional self-concept had a significant positive correlation with career decision-making self-efficacy (*r* = 0.693, *p* < 0.01) and a significant negative correlation with career decision-making difficulties (*r* = −0.496, *p* < 0.01).

### 3.3. Regression analysis and mediation effect test analysis

The results of the regression analysis are shown in Table 3. With demographic variables as controlled variables and career decision-making difficulties as the dependent variable, professional self-concept and career decision-making self-efficacy were taken as primary predictors in a multiple linear regression analysis. The results showed that professional self-concept could significantly positively predict career decision-making self-efficacy (*β* = 0.654, *p* < 0.01), and significantly negatively predict career decision-making difficulties (*β* = −0.470, *p* < 0.01). After the introduction of career decision-making self-efficacy, the influence coefficient (*β* = −0.217, *p* < 0.01) of professional self-concept on career decision-making difficulties decreased. It indicated that career decision-making self-efficacy is a partial mediator in the relationship between professional self-concept and career decision-making difficulties.

**Table 3:** Regression analysis of the relationship between variables (*n* = 276).

Outcome variable	Predictor variable	*β*	SE	*t*	*p*	LLCI	ULCI	*R* ^2^	*F*
CDMSE	PSC	0.654	0.071	13.777	0.000	0.837	1.116	0.393	189.806
CDDQ	PSC	−0.470	0.097	−8.711	0.000	−1.032	−0.651	0.203	75.878
CDDQ	CDMSE	−0.387	0.119	−3.259	0.001	−0.622	−0.154	0.286	60.241
	PSC	−0.217	0.079	−5.912	0.000	−0.619	−0.310		

CDDQ, career decision-making difficulties; PSC, professional self-concept; CDMSE, career decision-making self-efficacy.

**Figure 1:**
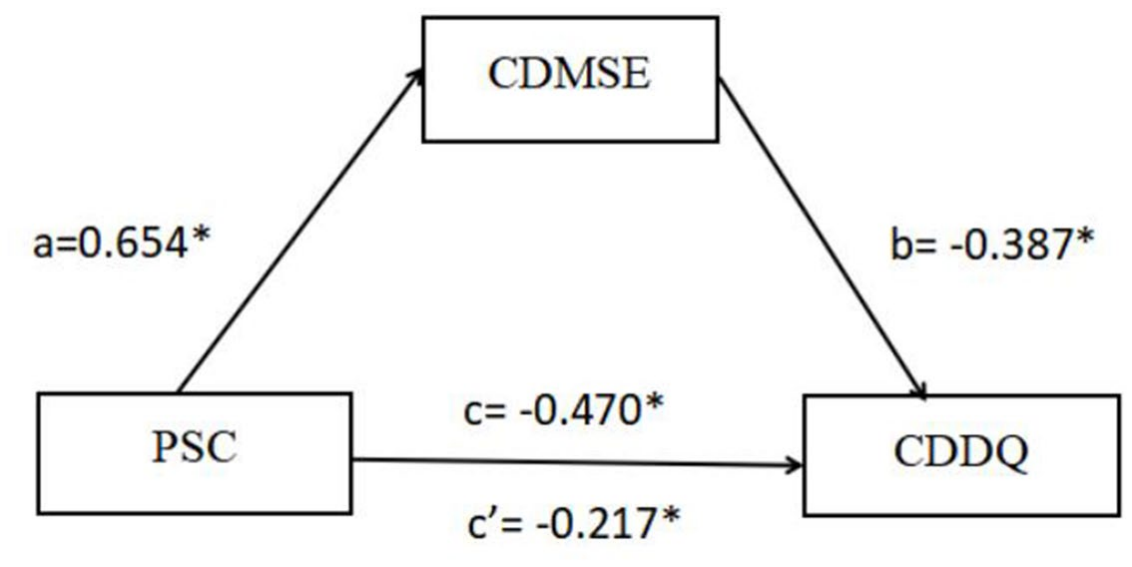
Model of the mediating role of CDMSE between PSC and CDDQ (*n* = 276) Career Decision-making Difficulties (CDDQ), Professional Self-concept (PSC), Career Decision-making Self-efficacy (CDMSE). *p < 0.01. a, the standardized regression coefficient between PSC and CDMSE; b, the standardized regression coefficient of CDMSE on CDDQ; c, the total effect between PSC and CDDQ; c’, the direct effect of PSC on CDDQ.

The results of the mediation effect test analysis are displayed in Table 4. Model 4 in the SPSS macro program PROCESS 4.0 developed by Hayes was used to test the mediating effect. After demographic variables were controlled, professional self-concept was used as the independent variable, career decision-making difficulties as the dependent variable, and career decision-making self-efficacy as mediating variables, and the Bootstrap method was used to calculate 95% confidence intervals for each of the 5,000 repeated draws. Result of the mediating effect analysis showed that Bootstrap’s 95% CI of total indirect effect did not contain 0 [Bootstrap 95% CI: −0.349, −0.156], accounting for 53.82% of the total effect. A visualization of the model is displayed in Figure 1.

**Table 4:** The mediating effect of career decision-making self-efficacy between professional self-concept and career decision-making difficulties (*n* = 276).

Path	Effect	Boot SE	Boot LLCI	Boot ULCI	Relative effect
Total effect	−0.470	0.097	−1.032	−0.651	100%
Direct effect	−0.217	0.119	−0.622	−0.154	46.18%
Indirect effect	−0.253	0.048	−0.349	−0.156	53.82%

## 4. Discussion

This study understands the current situation of career decision-making difficulties of postgraduate nursing students in China and explores the mediating role of career decision-making self-efficacy between professional self-concept and career decision-making difficulties based on SCCT. The results showed that professional self-concept and career decision-making self-efficacy were negative predictors of career decision-making difficulties, and career decision-making self-efficacy played a partial mediating role between professional self-concept and career decision-making difficulties. The results of this study can provide a basis for the employment guidance of postgraduate nursing students, to reduce their career decision-making difficulties and improve the level and quality of career decision-making.

### 4.1. The career decision-making difficulties of postgraduate nursing students are at a medium to high level

Our study showed that the total score of 2.84 (SD = 0.54) for career decision-making difficulties among postgraduate nursing students was moderately high, which was higher than the findings for undergraduate nursing interns (Yin and Dong, 2019) and for medical students (Zhu et al., 2021). The analysis may be due to the following reasons: In China, due to traditional beliefs and social opinions, nurses do not have a high social status, and many students choose nursing because of professional transfer. Therefore, they tend to be passive in their final career decisions. Secondly, nursing is a highly specialized discipline, and postgraduate nursing students have two main employment directions, becoming a teacher or a nurse, with a narrow range of employment. Most postgraduate nursing students prefer to be employed in schools, but most schools require applicants to have a doctoral degree. For healthcare organizations, the focus is not only on academic qualifications but also on clinical practice skills. While postgraduate nursing students have a great advantage in academic research and theoretical knowledge, clinical practice skills may not be superior to those of undergraduate students. Moreover, as job seekers, postgraduate nursing students have overly high expectations for career development prospects, salary, working environment, prestige, and social status, which are in strong contrast to reality. Overall, the career decision-making difficulties of postgraduate nursing students is prominent and requires active measures by relevant departments. From the three levels of society, school, and family, based on giving full understanding and concern to nursing postgraduates, we should strengthen their employment guidance, guide them to establish a correct career view, and help them understand themselves and their employment situation, to make satisfactory and conducive to the long-term development of career decision-making.

In addition, the current study showed that among the three dimensions of career decision-making difficulties, the lake of readiness dimension scored the highest, which was consistent with the results of previous studies (Yin and Dong, 2019; Zhu et al., 2021). Since these difficulties may inhibit the initiation of the career decision-making process, there may be more negative career-related outcomes before graduation if students do not deal with these difficulties in the early stages of their education (Viola et al., 2017; Kulcsar et al., 2020). Consistent with previous research (Schnoes et al., 2018), it is not surprising that postgraduate nursing students with experience in a career development program have lower levels of career decision-making difficulties. Career development skills acquired during the course can enhance students’ confidence in career exploration and decision-making. However, only 18.47% of the subjects in this study had this experience. Therefore, Nursing educators should create awareness of early career education, pay attention to early career education of postgraduate nursing students, build a complete and standardized career counseling curriculum, and add career scenario simulations inside and outside the curriculum to help postgraduate nursing students perform career preparation activities and make sound career decision-making.

### 4.2. The positive professional self-concept can reduce the career decision-making difficulties of postgraduate nursing students

The results of this study revealed that the professional self-concept of postgraduate nursing students was negatively related to career decision-making difficulties and had a negative predictive effect on career decision-making difficulties (*p* < 0.01), indicating that the more positive professional self-concept of postgraduate nursing students, the lower their career decision-making difficulties. Career development theory states that professional self-concept plays an important role in the career decision-making process and is a core element of career value orientation (Stephenson and Bell, 2019), and those with positive professional self-concept usually have a stronger sense of career competence and career beliefs, which leads to better preparation and more active collection of employment information when choosing a career, and then making effective career decision-making (Lv et al., 2014). It is suggested that nursing educators should pay attention to the cultivation and development of positive professional self-concept of postgraduate nursing students, and can help postgraduate nursing students to correctly understand career benefits and career values and strengthen their sense of career identity through mentorship (OARC, 2013) and peer counseling (Besnilian et al., 2016), to cultivate their positive professional self-concept, reduce the degree of career decision-making difficulties, and improve the level and quality of career decision- making.

### 4.3. Career decision-making self-efficacy played a partial mediating role between professional self-concept and career decision-making difficulties

The results of this study indicated that career decision-making self-efficacy among postgraduate nursing students was negatively associated with career decision-making difficulties, which is consistent with the findings of several previous studies (Storme et al., 2017; Santos et al., 2018; Jemini-Gashi et al., 2019). Furthermore, as shown in Table 4, career decision-making self-efficacy partially mediated the effect of professional self-concept on career decision-making difficulties (*p* < 0.01), suggesting that professional self-concept can not only directly influence career decision-making difficulties, but also indirectly through career decision-making self-efficacy. This further confirms the important contribution of the career choice efficacy intervention to career decision-making and career development. According to Bandura’s self-efficacy theory (Bandura, 1977), career decision-making self-efficacy is an individual’s self-perception of his or her abilities, career interests, and career aspirations during the career choice process, and this level of self-perception influences the individual’s thinking style, emotional response, effort, and career choice, etc. SCCT (Lent et al., 1994) stated that success and failure experiences related to career activities, supportive role models, social and verbal persuasive communication, and positive emotional responses are the main sources of information that influence the formation and development of career decision-making self-efficacy. Therefore, the relevant departments of the university can enhance the career decision-making self-efficacy by inviting outstanding nursing seniors and alumni to conduct lectures related to disciplinary development and employment, so that postgraduate nursing students can feel the sense of achievement, honor, and pride brought by nursing work. In addition, the formation of career decision-making self-efficacy is influenced by factors such as attribution style, goal setting, and timely feedback (Betz, 2004). So, nursing educators should guide postgraduate nursing students to face difficulties and setbacks with an optimistic attitude, instruct them to use correct attribution styles, and actively seek social support from all aspects to improve career decision-making self-efficacy and make effective career decision-making.

## 5. Limitations

The present study has several limitations. Firstly, this study is a cross-sectional study, and it is not possible to directly infer causal relationships between variables. Secondly, all data were collected through a self-report questionnaire, which may lead to some bias in the study results. Thirdly, the sample size was small, which limited the generalizability of the results. Future sample sizes could be increased and a longitudinal study could be conducted to explore in-depth other influencing factors and targeted interventions for career decision-making difficulties among postgraduate nursing students. This will provide nursing educators with ways to improve the career decision-making difficulties of postgraduate nursing students.

## 6. Conclusion

In conclusion, the career decision-making difficulties of postgraduate nursing students are at a medium to high level for postgraduate nursing students, and professional self-concept and career decision-making self-efficacy were negatively correlated with career decision-making difficulties, and career decision-making self-efficacy mediated between professional self-concept and career decision-making difficulties. It is demonstrated that measures and strategies to improve career decision-making self-efficacy are expected to mitigate the effects of professional self-concept on career decision-making difficulties. It is suggested nursing educators need to develop a complete and standardized career counseling curriculum for postgraduate nursing students, and should pay attention to the cultivation and development of positive professional self-concept and career decision-making self-efficacy of postgraduate nursing students to reduce their career decision-making difficulties and help them make effective career decision-making.

## Data availability statement

The raw data supporting the conclusions of this article will be made available by the authors, without undue reservation.

## Ethics statement

The studies involving human participants were reviewed and approved by the First Affiliated Hospital of Chongqing Medical University (No. 2022-10). The patients/participants provided their written informed consent to participate in this study.

## Author contributions

YB, SM, GW, and ML made significant contributions to the conception and design, acquisition of data, or analysis and interpretation of data, participated in drafting the article or critically revising important intellectual content, agreed to submit the article to the current journal, gave final approval of the version to be published, and agreed to take responsibility for all aspects of the work. All authors contributed to the article and approved the submitted version.

## Conflict of interest

The authors declare that the research was conducted in the absence of any commercial or financial relationships that could be construed as a potential conflict of interest.

## Publisher’s note

All claims expressed in this article are solely those of the authors and do not necessarily represent those of their affiliated organizations, or those of the publisher, the editors and the reviewers. Any product that may be evaluated in this article, or claim that may be made by its manufacturer, is not guaranteed or endorsed by the publisher.
